# Comparing Extraction Methods for Biomarker Steroid Characterisation from Soil and Slurry

**DOI:** 10.1007/s11270-020-04871-w

**Published:** 2020-10-09

**Authors:** Amber Manley, Adrian L. Collins, Adrian Joynes, Per-Erik Mellander, Phil Jordan

**Affiliations:** 1 Sustainable Agriculture Sciences, Rothamsted Research, North Wyke, Okehampton, EX20 2SB UK; 2 Agricultural Catchments Programme, Teagasc, Environment Research Centre, Johnstown Castle, Co., Wexford, Ireland; 3 School of Geography and Environmental Sciences, Ulster University, Cromore Road, Coleraine, BT52 1SA UK

**Keywords:** Lipids, ASE, Bligh and Dyer, Soxhlet, Faecal pollution

## Abstract

Clean water is a precious resource, and policies/programmes are implemented worldwide to protect and/or improve water quality. Faecal pollution can be a key contributor to water quality decline causing eutrophication through nutrient enrichment and pathogenic contamination. The robust sourcing of faecal pollutants is important to be able to target the appropriate sector and to engage managers. Biomarker technology has the potential for source confirmation, by using, for example the biomarker suite of steroids. Steroids have been used in the differentiation of human and animal faeces; however, there is no unequivocal extraction technique. Some of the methods used include (i) Soxhlet extraction, (ii) Bligh and Dyer (BD) extraction, and (iii) accelerated solvent extraction (ASE). The less costly and time intensive technique of ASE is particularly attractive, but a current research gap concerns further comparisons regarding ASE lipid extraction from soils/slurries compared with the more traditional Soxhlet and BD extractions. Accordingly, a randomised complete block experiment was implemented to assess differences between the three extraction methods, differences between the different sample types, and the interactions between these two factors. Following GC-MS, it was found that there was no significant difference between the results of the steroid extraction methods, regardless of the type of sample used, for the quantity of each steroid extracted. It was concluded that ASE could be used confidently instead of the more established steroid extraction methods, thereby delivering time and cost savings.

## Introduction

Faecal matter transferred through and exported from catchments, originating from various point and diffuse sources, can be a vector for phosphorus (P) and nitrogen (N) causing eutrophication in water bodies and causing human health pressures due to associated pathogens (Jang et al. 2017). Determining the source proportions of faecal matter is challenging and biomarker technologies have been applied in recent years to address this (Unno et al. 2018). Biochemical methods have also been shown to have potential applications in tracing nutrients, organic matter and fine-grained sediments from these sources, and particularly in agricultural catchments (Mudge and Duce 2005; Arnscheidt et al. 2007).

Steroids are a potential lipid biomarker suite used in previous studies (Δ5-sterols, stanols, stanones) (Prost et al. 2018) and are an important class of organic molecules present in most cells (Huang and Meinschein 1976). The ability to use steroids as biomarkers of faecal pollution was developed by Leeming et al. (1994, 1996, 1997, 1998) and used as robust discriminators between human and herbivorous faecal matter based on their differing distributions. Using this fingerprinting approach, steroids have been used to confirm sources of faecal contamination including wastewater outputs (Grimalt et al. 1990; Leeming et al. 1997; Mudge et al. 1999), farm land slurry applications (Jardé et al. 2009), or animal waste runoff (Nash et al. 2005; Tyagi et al. 2007). Despite this utility, there is no unequivocal universally applied technique for isolating lipid biomarkers prior to mass spectrometry analysis. Instead, a wide range of extraction procedures is applied in contemporary practice, including Soxhlet extraction, Bligh and Dyer (BD) extraction, and accelerated solvent extraction (ASE). An overview is provided here.

The Soxhlet extractor was developed by Franz von Soxhlet in 1879 as laboratory equipment to be used in the extraction of lipids and has become one of the most well-established lipid extraction techniques used (Lyons et al. 2015; Kolm et al. 2018). In fact, the Soxhlet extraction method has been used as the primary reference to which other, newer extraction methods have been compared over the several decades (Luque de Castro and Priego-Capote 2010). Whilst being a robust and well-established technique and has other advantages, the method has disadvantages. One advantage is the continuous cycle that the solvent undergoes (evaporation–condensation) that brings the solid sample in contact with fresh solvent and facilitates extraction. Secondly, the equipment to perform Soxhlet extraction is relatively cheap (excluding the solvent costs). Thirdly, the methodology is quite simple and requires little training (Luque de Castro and Priego-Capote 2010). The main disadvantages include lengthy extraction times and larger extractant sizes that result in more waste (which has both economic and environmental implications). Difficulties with automation and a lack of agitation are other disadvantages as well as the potential for thermal decomposition of the sample due to the high temperatures used (Luque de Castro and Priego-Capote 2010).

There have been various alterations made to conventional Soxhlet extraction over the years that aim to address the limitations. These include high pressure Soxhlet extraction wherein high pressure is achieved by placing the extractor in a cylindrical autoclave (Ndiomu and Simpson 1988) or by the use of supercritical fluid–Soxhlet extractors (Luque de Castro and Priego-Capote 2010), ultrasound-assisted Soxhlet extraction (makes use of an ultrasonic probe being added to the sample chamber; Luque-Garcıa and De Castro 2004), and microwave-assisted Soxhlet extraction (use of microwave irradiation on the sample chamber) (Luque de Castro and Priego-Capote 2010). Each acts on the advantages and disadvantages of conventional Soxhlet extraction, but adds complexity, cost, and technical training.

Another similarly well-established method is the BD extraction. The BD method was developed in 1959 (Bligh and Dyer 1959) for extracting lipids, and has become the standard procedure to separate total lipid fractions from samples. It is both less time consuming and less costly than Soxhlet extraction. BD has been used in several areas such as in hospitals, pharmaceuticals, and food studies (Breil et al. 2017). However, BD makes use of chemicals like chloroform (toxic and carcinogenic) giving the method a safety disadvantage that makes it difficult for large scale use (Breil et al. 2016). As such, there are some limitations to its applicability (Hussain et al. 2014; Breil et al. 2017). Any improvements to BD have focused mostly on replacing the solvents used in the original method to less dangerous alternatives (Grima et al. 1994; Lee et al. 1998; Sheng et al. 2011; Caprioli et al. 2016). However, the methods employed remain toxic to both humans and the environment (though lessened using fume cupboards). Various methodological alterations have also been investigated to help improve BD. Among them are the use of ultrasound, microwaves, heat, pressure, or beads (Axelsson and Gentili 2014; Berndmeyer et al. 2014; Ryckebosch et al. 2012; Teo and Idris 2014; Medina et al. 2015; Lee et al. 2010; Cescut et al. 2011). Whilst both Soxhlet and BD methods have improved over time, both methods are still timely and work intensive.

A promising alternative is the ASE technique (Richter et al. 1996; Jansen et al. 2006). In summary, ASE extracts samples under elevated temperature, whilst elevated pressure ensures that volatile extractants remain liquid. ASE can be completely automated; it employs very small extractant volumes (normally 5–30 mL) and has typical extraction times of less than an hour (Richter et al. 1996; Jansen et al. 2006). As such, the technique has the potential to overcome the main disadvantages of both Soxhlet and BD extraction methods. This reduction in labour time and solvent can result in ASE being cheaper to use per sample than both alternative methods. However, ASE requires specific instrumentation unlike Soxhlet and BD (that uses glassware) that can cost approximately £30,000 (cost estimated in 2019). In some circumstances, compared with these conventional methods, modern ASE methods have been able to yield equivalent, if not better, extraction efficacies (Jansen et al. 2006; Balasubramanian et al. 2013). However, whilst the use of ASE to extract organic contaminants from media such as soils is now reasonably well-established (Giergielewicz-Mozajska et al. 2001; Chitescu et al. 2012), its application to the specific extraction of lipids from other solid matter has received less attention. A comparison of ASE with more established techniques is important if ASE is to be used in biomarker studies that require mass spectrometry analysis on potentially high sample numbers. This is because differences in extraction efficiencies for several types of lipids between ASE and other techniques would lead to a difference in the composition of the biomarker signature that is obtained.

Therefore, the aim of the current study was to examine and compare the efficiency of ASE with more established Soxhlet extraction and BD extraction methods, for extracting typical lipid biomarkers, including steroids, from both animal slurry and soil samples. These two biomarker sources, individually and in combination, were characterised here as potential diffuse sources of faecal matter in agricultural catchments.

## Materials and Methods

### Study Area and Experimental Design

Higher Wheaty, a grazed grassland field at Rothamsted Research North Wyke, UK, was used in this experiment. This field has been used for livestock grazing and has had applications of fertilisers/herbicides in the last four years prior to sampling. Soil belongs to the Halstow soil series—a clayey typical noncalcareous pelosol in head from clay shale. Preliminary laboratory test results of the soil in 2018, shown in Table 1, indicate that the pH and P levels are higher than expected, but this is likely due to a large application of P_2_O_5_ on to the field in 2016. The cattle slurry used in this experiment was collected from an open air slurry lagoon on a nearby dairy farm in SW England on a grazed grassland system with winter housing of animals.

**Table 1:** Preliminary laboratory test results for the Higher Wheaty study field, Rothamsted Research North Wyke, Devon, completed in April 2018. Soil P (and K, Mg) is based on the Olsen extraction method and places the soil at just above the agronomic optimum (index 3 > 26 mg/L)

Area (ha)	pH value	P (index)	P (mg/L)	K (index)	K (mg/L)	Mg (index)	Mg (mg/L)
0.99	6.9	3	27.4	1	108	2	97

**Fig. 1:**
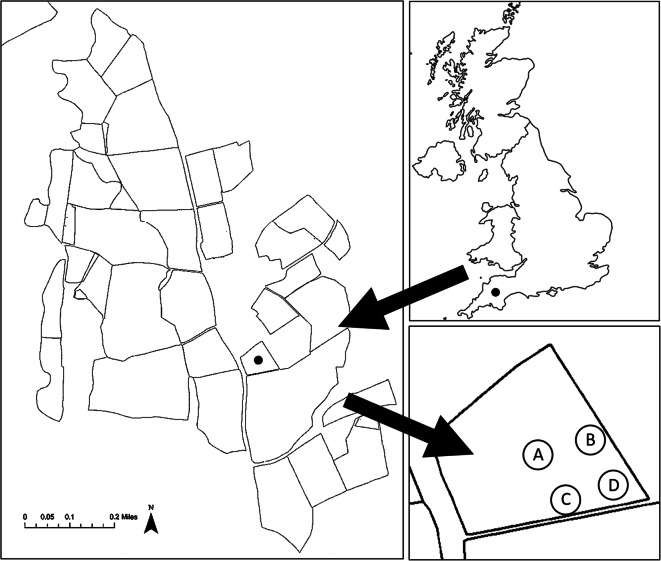
An image of the sampling sites in Higher Wheaty field, Rothamsted Research North Wyke, UK (black circles marked on the maps). Samples were taken from four sites in the study field. These sites comprised areas of the field where some natural variation would be expected. Plot A is mid-field, so most likely exposed to higher levels of animal wastes in the past. Plot B is by the edge of the field where mostly grass grows. Plot C is similarly on the edge of the field but has a mixture of grass and weeds growing. Plot D is by the gate to the field, so the soil is likely to be more compacted. This was done to provide a wide range of values for checking agreement regarding differences between methods and sample types

**Fig. 2:**
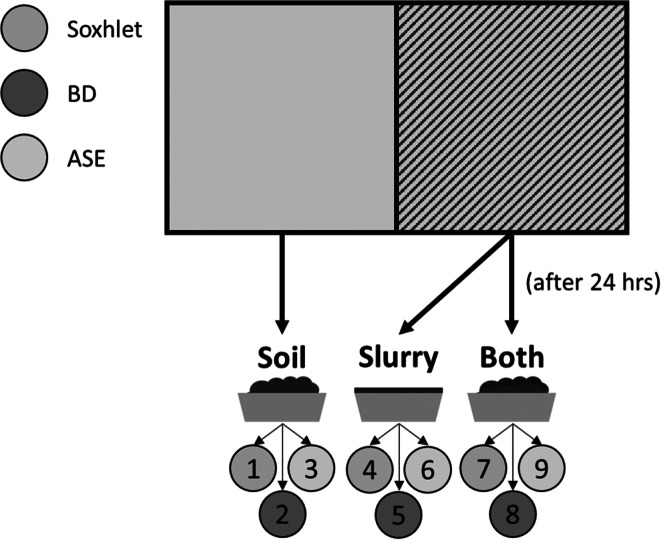
A visual representation of the plots and sampling used in this experiment. The non-patterned side of the plot was on the most downhill side of the entire plot to prevent the soil sampling from affecting later sampling. Only soil came from this side. The patterned side was on the most uphill side of the plot and had 610 mL of slurry applied and was left for 24 h before sampling. Slurry and both (soil with an application of slurry) was taken from this side. Each of these sample types were extracted using all three extraction methods. This made it so that each plot (A, B, C, and D from Fig. 1) had nine samples in a block, i.e. A1–9, B1–9, C1–9 and D1–9

A randomised complete block design was created to assess for differences between the extraction methods, differences between the different sample types, and the interactions between these two factors. Three types of sample were collected for this study, i.e. slurry (*Slurry*), topsoil of the plot to a depth of 2 cm prior to the slurry application *(Soil*), and the topsoil 24 h after the slurry application (*Both*). This design utilised four 0.6 m^2^ plots to act as replicates for each sample type taken from different areas of the same field, as shown in Fig. 1. These plots were each split in half with soil sampled from the downhill half and slurry spread only on the uphill half then sampled 24 h later. These four replicates of each sample type were then divided into three sub-samples each; one to be analysed for each extraction method per replicate, per sample type, as shown in Fig. 2. This was designed so replicates would contain relatively homogenous material and so provide a fair comparison of the three extraction methods. As the samples taken from each plot were linked (soil pairs of before and after slurry application), the plots could be considered a block of nine samples (three sample ‘Types’ to be analysed by three different steroid extraction ‘Methods’).

### Chemical Preparation and Extraction Methods

All equipment used in the study was washed thoroughly prior to use. Aluminium foil and containers were muffle furnaced (450 °C for at least 4 h) to remove organic contaminants before use. All glassware was washed thoroughly and rinsed in acetone before use. The custom-made 2 cm depth soil corer was washed in Virkon disinfectant, MilliQ water, and acetone prior to use. Between samplings in the field, equipment was washed with Virkon disinfectant and MilliQ water to minimise contamination. Sieves and grinders were similarly washed thoroughly with Microsol^4^ disinfectant, MilliQ water, and grinders also received an industrial methylated spirit (IMS) wash both prior to, and between, sample use to avoid cross-contamination.

For each block, cores were taken and loosely sealed in an aluminium foil container. Following soil sampling, 610 mL of slurry was applied manually to the un-cored half of the block at a rate of 3.3 L/m^2^. This rate is to simulate a typical slurry application rate of 33 cubic metres of slurry per hectare (Brennan et al. 2012). The slurry samples remaining were saved and transferred to an aluminium container in the laboratory. The samples were then uncovered and placed in an oven set at 30 °C to dry. After 24 h, the soil with slurry (both) samples were collected and then placed in the same oven set at 30 °C to dry.

All samples were considered dry once a constant weight was achieved. Once dry, samples were sieved to 2 mm to remove any large debris, finely milled, and stored in glass vials. Samples were analysed as block composites (i.e. all samples from one block at a time) to account for any possible variation arising from longer latency periods between storage and analysis. Each block analysis amounted to three samples split between the three extraction methods (three extractions to be completed per method, per block, equalling nine samples/analyses per block), shown in Fig. 2. Each extraction method commenced at the same time. For all extractions, 5 g of soil/both was used and 0.5 g of slurry (Leeming et al. 1996). These values were chosen to ensure that the optimum amount of steroid was extracted for analyses (with slurry requiring much less sample due to the inherently greater levels of steroids). The internal standard (100 μL of 0.2 mg/mL 5β-pregan-3α-ol) that the steroids were quantified against (concentrations relative to the standard) was placed directly with the soil/slurry sample in the thimble/vial/cell. Each batch of extractions had analytical blanks that were analysed following the same procedure as the samples to check for contamination. For this purpose, the internal standard was added to an empty pre-cleaned Soxhlet thimble, BD solvents, and empty ASE cell with no sample.

### Soxhlet Extraction

Prior to extraction, the cellulose Soxhlet thimbles and cotton wool tops used to contain the samples in the extraction chamber were cleaned by pre-extracting with 250 mL of dichloromethane (DCM):acetone (9:1) for at least 6 h in the Soxhlet apparatus. The thimble was then removed and allowed to dry. The methodology for extraction is described in Puttock et al. (2014) and was used by Norris et al. (2013). A thimble containing the sample (topped with cotton wool) and the internal standard was placed in the extraction chamber and extracted with 250 mL of DCM:acetone, (9:1) for 24 h. After extraction, the total lipid extract (TLE) was rotary evaporated, re-suspended, and transferred to a vial in 3 × 2 mL of DCM:acetone (1:1) and finally evaporated at 37 °C under a gentle stream of N_2_.

### Bligh and Dyer Extraction

All the solvents required for BD extraction were prepared just before use. Extraction began by adding 10 mL of the BD solvent (BDS:methanol, chloroform, and buffered water) and the internal standard to the sample in a culture tube. This was then vortexed, ultrasonicated for 15 min, centrifuged at 2500 rpm for 5 mins, and then the supernatant collected. This was repeated three more times (using 5 mL, then 3 mL twice BDS instead). After this, 5 mL of chloroform and 5 mL of DCM extracted water was added to the supernatant. This was then vortexed, centrifuged (2500 rpm for 5 min), and then the organic phase collected. This was repeated three times (except for additional DCM extracted water and using 3 mL of chloroform instead). After extraction, the TLE was rotary evaporated, re-suspended in 3 × 1 mL of DCM:acetone (1:1) and transferred to a vial. Any water was removed from the extract by running it through a drying column (Pasteur pipette plugged with Soxhlet cleaned cotton wool and filled with dried sodium sulphate). The column was washed with 3 mL of DCM:acetone (1:1) prior to the addition of the extract. The extract was then chased by 1 mL of DCM:acetone (1:1), collected with the sample, and then evaporated under a gentle stream of N_2_.

### Accelerated Solvent Extraction

A Dionex ASE 350 (Thermo Scientific) was used in this study. Firstly, the stainless steel ASE cells were cleaned by being rinsed in DCM and then allowed to dry. A cellulose cell filter (27 mm) was then placed in the centre of the PEEK ring and pressed inside. The sample was then placed in the cell along with the internal standards. A solvent mixture of DCM:acetone (9:1) was used. The automatic extraction process comprised the following steps: (i) extraction vessels with biomass samples were loaded into the extractor; (ii) cells were filled with solvents up to a pressure of 1500 kPa; (iii) extraction vessels were heated for 5 min; (iv) two static cycles of each 5 min; (v) the vessel was rinsed using extraction solvent; and (vi) solvent was purged from the vessel with N_2_ gas for 100 s. The extracts were then retained in 60 mL collection vials. After extraction, the TLE was rotary evaporated, re-suspended in 3 × 2 mL of DCM:acetone (1:1), transferred to a vial and finally evaporated under a gentle stream of N_2_.

### Analyses Following Extraction

To quantify the steroids, extracts were saponified to break any ester bonds and free all alcohols in the samples. This was performed using 2 mL of 5 M potassium hydroxide in 90% methanol and heated at 100 °C for 1 h. Once cooled, 2 mL of DCM extracted water was added to the organic extract, which was then acidified using 6 M hydrochloric acid. Saponified organics were then extracted into chloroform (3 × 2 mL), combined, and blown down. All extracts then underwent drying and fractionation (consequently) in columns using glass Pasteur pipettes, cleaned cotton wool (to plug), and 5 cm of either activated sodium sulphate (drying column) or activated silica gel (flash column). Both types of column were cleaned using DCM prior to sample addition. To elute the extracts, 1 mL of DCM was added to drying columns and 5 mL of DCM, then 5 mL of DCM:methanol (1:1) (second fraction containing the steroids) to the flash columns. The extracts were collected and dried under a gentle flux of nitrogen.

Fractionated extracts were derivatised by silylation to improve the chromatography during GC-MS analysis. This was done by adding 50 μL of derivatizing agent (N,O-bis(trimethylsilyl)trifluoroacetamide [BSTFA] + trimethylchlorosilane [TMCS]) to samples which were then placed in a heating block at 70 °C for 1 h. Extracts were cooled and dried under a gentle flux of nitrogen. For analysis, extracts were suspended in 75 μL of hexane and transferred to a GC-MS vial ready for analysis. The samples were analysed by a methodology derived from Bull et al. (2003) using an Agilent Technologies 6890GC/5973N GC-MS with 7683 autosampler. The samples were separated on an Agilent HP-5 ms, 30 m × 250 μm × 0.25 μm column with an oven temperature programme as follows: hold for 1 min at 40 °C, then increase from 40 °C to 230 °C at a rate of 20 °C min^−1^, then to 300 °C at a rate of 2 °C min^−1^, and finally hold for 15 min. The source was at 230 °C and quadrupole at 150 °C, with scanning from m/z 29–550 at electron voltage 70 eV. The total ion count (TIC) data were acquired and analysed using Agilent Chemstation software. The biomarkers were identified by using known characteristic spectra and comparing with those in the National Institute of Standards spectral library (NIST, US Gov.). The TIC data were quantified against their relevant internal standard and had a detection limit of 0.3 μg/g. The internal standard here is used specifically to determine the analyte concentration and not to monitor the internal standard recovery.

### Statistical Analyses

To compare the extraction methods, the data were analysed in Genstat 19. Any values of 0 were due to the steroidal level being below the level of detection. As such, to continue with statistical analyses, these values were changed to 0.15 μg/g (the value halfway between 0 and the detection limit). Due to innate differences between the samples, the data were log transformed prior to all analyses. For ANOVA, steroids were compared using the block number and sample and sub-sample number as a block, and the method used and the type of sample as a treatment. Multivariate analysis was also completed to simultaneously consider the differences between treatments across the variables. Principal component analysis (PCA) was then performed to assess the variation in the data using the correlation matrix. This was followed by canonical variate analysis to identify the key variables contributing to these differences, using both method and sample type individually as grouping factors. The significance level for all statistical tests was 0.05 and the degrees of freedom for all tests of type is F_2,6_, for method is F_2,18_, and for type method is F_4,18._

## Results

The steroids identified in this study are presented in Table 2 together with their systematic and trivial names and class. A total of 15 steroids were identified in the 12 slurry, soil, and both samples. This includes the precursor steroid cholesterol and key faecal biomarkers coprostanol and 24-ethyl-coprostanol. In summary, there were five derivatives of cholesterol including the precursor itself, one C27 bile acid/alcohol/derivative, seven stigmasterols/C24-ethyl derivatives, and two ergosterols/C24-methyl derivatives.

**Table 2:** A list of the steroids identified in the samples, with both common and systematic names, formula, chemical class, and subclass according to the LIPID MAPS initiative (Fahy et al. 2009). *ST01*, sterol; *ST04*, bile acids and derivatives; *ST0101*, cholesterol and derivatives; *ST0403*, C27 bile acids, alcohols, and derivatives; *ST0104*, stigmasterols and C24-ethyl derivatives; *ST0103*, ergosterols and C24-methyl derivatives. A x* by the name of the steroid indicates that it was not found on the database and thus interpreted separately using the same categories

Common name	Systematic name	Formula	Main class	Sub class
Coprostanol	5β-Cholestan-3β-ol	C_27_H_48_O	ST01	ST0101
Epi-Coprostanol	5β-Cholestan-3α-ol	C_27_H_48_O	ST04	ST0403
Cholesterol	Cholest-5-en-3β-ol	C_27_H_46_O	ST01	ST0101
5α-Cholestanol	5α-Cholestan-3β-ol	C_27_H_48_O	ST01	ST0101
5β-Campestanol*	24α-Methyl-5β-cholestan-3β-ol	C_28_H_50_O	ST01	ST0104
Epi-5β-Campestanol*	24α-Methyl-5β-cholestan-3α-ol	C_28_H_50_O	ST01	ST0104
24-Ethyl coprostenol*	24β-Ethyl-Δ22-coprostenol	C_32_H_58_OSi	ST01	ST0101
Epi-24-ethyl coprostenol*	24α-Ethyl-Δ22-coprostenol	C_32_H_58_OSi	ST01	ST0101
24-Ethyl-coprostanol	5β-Stigmastanol	C_29_H_52_O	ST01	ST0104
Epi-24-ethyl-coprostanol	Epi-5β-Stigmastanol	C_29_H_52_O	ST01	ST0104
Campesterol	Campest-5-en-3β-ol	C_28_H_48_O	ST01	ST0103
Campestanol	5α-Campestan-3β-ol	C_28_H_50_O	ST01	ST0103
Stigmasterol	Stigmasta-5,22E-dien-3β-ol	C_29_H_48_O	ST01	ST0104
β-Sitosterol	(8S,9S,10R,13R,14S,17R)-17-[(1R,4R)-4-Ethyl-1,5-dimethyl-hexyl]-10,13-dimethyl-2,3,4,7,8,9,11,12,14,15,16,17-dodecahydro-1H-cyclopenta[a]phenanthren-3-ol	C_29_H_50_O	ST01	ST0104
Stigmastanol	5α-Stigmastan-3β-ol	C_29_H_52_O	ST01	ST0104

The cattle slurry used in this study on average contained a total of 3500 μg/g of steroids, soil 30 μg/g, and both 35 μg/g. In general, the slurry samples contained higher quantities of each steroid identified than in the soil or both samples (Table 3). The predominant steroids in the slurry samples were 24-ethyl-coprostanol and epi-24-ethyl-coprostanol (by at least 3 times the next largest). The steroids, β-sitosterol and stigmastanol, were predominant in soil and both samples (2 and 6 times, respectively).

**Table 3:** Steroidal means for the treatment combinations (μg/g), together with their standard error of differences (SED); 0.3 μg/g represents the detection limit

Steroid	Method	Type means			Type method SED	SED except when comparing means with the same level(s) of type
		Slurry	Soil	Both		
Coprostanol	ASE	285.71	0.75	0.69	0.24	0.23
	BD	290.70	0.47	1.00		
	Sox	294.55	0.16	0.66		
Epi-Coprostanol	ASE	70.92	0.23	0.22	0.20	0.14
	BD	66.73	0.17	0.37		
	Sox	75.50	0.10	0.31		
Cholesterol	ASE	159.70	2.26	1.36	0.23	0.26
	BD	138.07	2.18	1.33		
	Sox	171.27	1.79	4.01		
5α-Cholestanol	ASE	193.97	0.67	0.87	0.13	0.09
	BD	194.20	0.59	1.03		
	Sox	149.06	0.59	0.89		
5β-Campestanol	ASE	54.90	0.24	0.13	0.30	0.19
	BD	42.06	0.24	0.32		
	Sox	71.92	0.21	0.15		
Epi-5β-campestanol	ASE	148.29	0.42	0.36	0.35	0.40
	BD	28.74	0.31	0.59		
	Sox	151.57	0.20	0.45		
24-Ethyl soprostenol	ASE	100.34	0.70	1.01	0.15	0.10
	BD	100.29	0.87	0.95		
	Sox	104.51	0.80	0.81		
Epi-24-ethyl coprostenol	ASE	103.96	0.41	0.44	0.15	0.08
	BD	99.62	0.31	0.66		
	Sox	110.83	0.26	0.50		
24-Ethyl-coprostanol	ASE	737.60	2.18	2.09	0.25	0.19
	BD	737.29	1.69	3.06		
	Sox	760.43	1.36	1.27		
Epi-24-ethyl-coprostanol	ASE	760.95	2.68	1.12	0.33	0.24
	BD	716.90	1.03	2.66		
	Sox	788.28	0.38	2.29		
Campesterol	ASE	0.50	0.55	3.21	0.40	0.33
	BD	0.15	1.18	2.24		
	Sox	0.42	2.59	3.18		
Campestanol	ASE	213.06	0.92	1.02	0.09	0.03
	BD	201.65	0.71	0.97		
	Sox	218.65	0.75	1.01		
Stigmasterol	ASE	27.77	2.25	2.42	0.06	0.05
	BD	30.19	2.54	2.50		
	Sox	25.37	2.26	2.22		
β-Sitosterol	ASE	249.28	10.00	10.64	0.05	0.03
	BD	236.98	9.78	10.80		
	Sox	249.95	9.87	10.51		
Stigmastanol	ASE	642.81	4.60	4.98	0.10	0.02
	BD	617.22	3.57	4.22		
	Sox	665.26	4.21	5.15		

Whilst the steroid profiles of the soil and both samples were similar, there were three steroids that varied significantly between them. The quantity of coprostanol was almost twice as much in both than in soil samples. The steroid campesterol was not identified within slurry samples in three of the four blocks. Soil and both samples contained campesterol in all blocks. 5β-Campestanol was not found or only in trace amounts in three of the blocks for both and soil sample types (> 0.5 μg/g). In both instances, the block where campesterol (within slurry) and 5β-campestanol (in soil and both) were taken by the study field gate (Fig. 1).

Following ANOVA on the data, it was shown that all steroids showed a significant difference (*P* ≤ 0.05) regarding sample type (Table 4). The ANOVA for the steroids showed there to be no statistically significant difference (*P* = ≤ 0.05) between the method used to analyse the samples in all steroids except one (stigmastanol). This steroid showed a significant difference (*P* = < 0.001) between the amount of sample obtained between methods (Table 4). There was often considerable variation between the methods within these latter two types, whereas there was very little variation between methods for slurry (but the variation was still small, hence the non-significant interactions) (Table 4). Furthermore, there was no significant interaction effect between sample type and method except for in Epi-24-ethyl-coprostanol (Table 4). However, when block 1 (that contained unusually low values compared with the other blocks for this steroid) were removed and the ANOVA re-rerun, the mean values remained largely unchanged and the *F* values became 0.002 (type), 0.228 (method), and 0.079 (interaction).

**Table 4:** The results of the ANOVA for the steroid biomarkers, showing any significant differences between method, sample type, and if there was any interaction effect between the two. The variance ratio (*F* value) of each is also shown. Significant results (*P* = ≤ 0.05) are highlighted in italics

Steroid	Method *F* value	Method *P* value	Type *F* value	Type *P* value	Interaction effect *F* value	Interaction effect *P* value
Coprostanol	1.8	0.194	240.2	*< 0.001*	1.49	0.246
Epi-coprostanol	0.53	0.599	156.47	*< 0.001*	1.83	0.168
Cholesterol	0.67	0.524	358.33	*< 0.001*	0.86	0.509
5α-Cholestanol	0.89	0.427	295.52	*< 0.001*	0.51	0.73
5β-Campestanol	0.35	0.712	57.67	*< 0.001*	1.42	0.267
Epi-5β-campestanol	0.43	0.657	202.91	*< 0.001*	1.11	0.384
24-Ethyl coprostenol	0.14	0.872	194.49	*< 0.001*	0.45	0.771
Epi-24-ethyl coprostenol	0.61	0.555	201.98	*< 0.001*	2.8	0.057
24-Ethyl-coprostanol	1.23	0.317	108.93	*< 0.001*	0.75	0.572
Epi-24-ethyl-coprostanol	0.91	0.419	72.82	*< 0.001*	3.27	*0.035*
Campesterol	1.41	0.27	5.26	*0.048*	1.16	0.36
Campestanol	3.45	0.054	576.87	*< 0.001*	1.72	0.19
Stigmasterol	2.24	0.135	290.38	*< 0.01*	0.17	0.952
β-Sitosterol	0.15	0.861	593.47	*< 0.001*	0.24	0.912
Stigmastanol	15.28	*< 0.001*	307.42	*< 0.001*	2.31	0.097

**Fig. 3:**
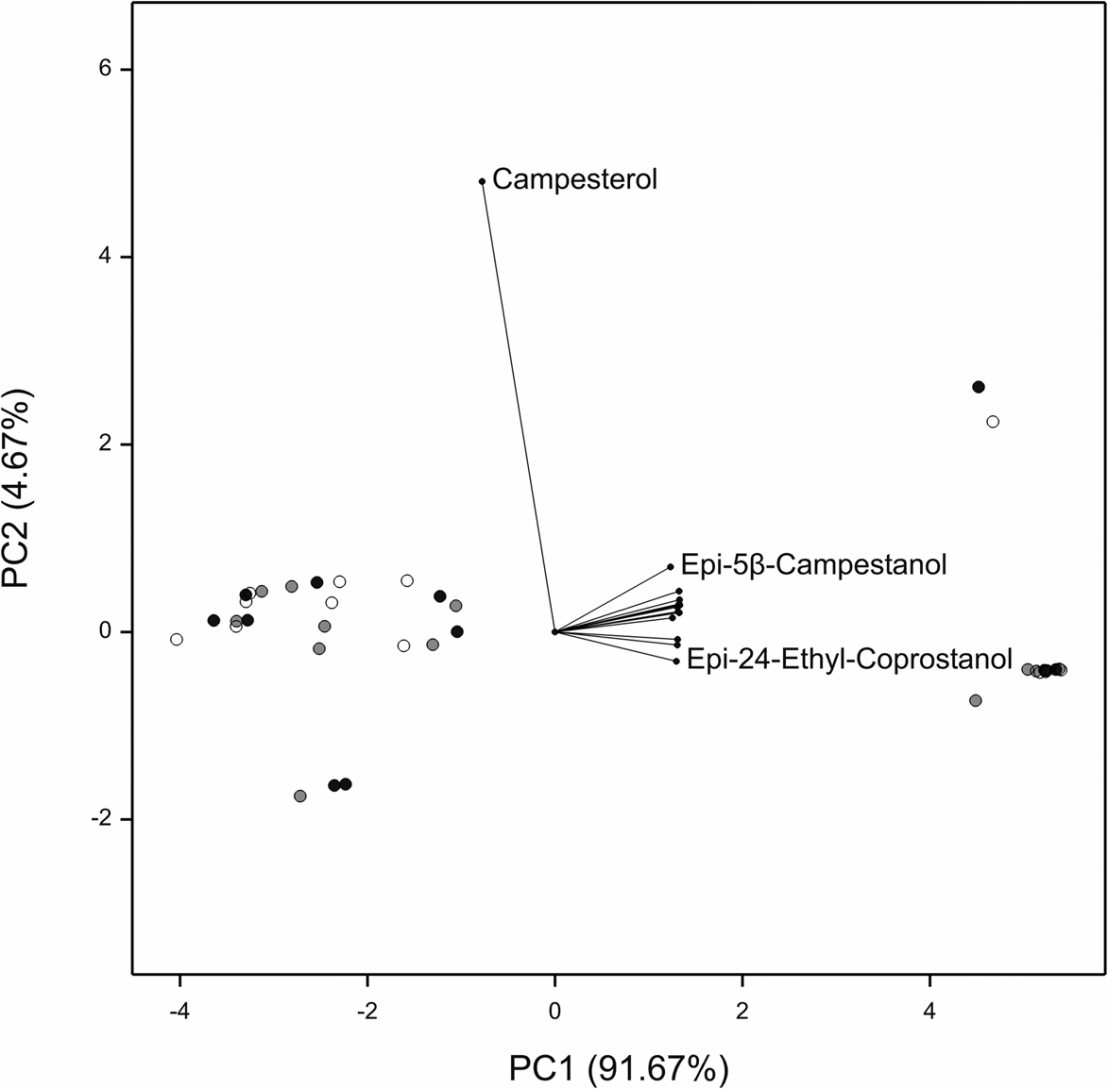
The principal components biplot for the steroidal analyses. The grouping factor was the method (ASE, black circle; Bligh and Dyer, grey circle; Soxhlet, white circle). Steroids: 5β-campestanol, coprostanol, 24-ethyl-coprostanol, epi-coprostanol, epi-24-ethyl-coprostenol, 5α-cholestanol, campestanol, stigmastanol, stigmasterol, 24-ethyl-coprostenol, cholesterol, and β-sitosterol are not labelled due to the proximity of their markers

To further investigate the relationships between the methods or types used, PCA was performed. The first 2 principal components (PC) explained 96.34% of the total variation present across all the steroids (further PCs were not included as their contribution was small). The loadings in the PCA output (Table 5) indicate the importance of the different variables with regards to the overall variability. PC1 is the linear combination (a weighted sum) of the variables that explains the largest proportion of the variation in the data. Similarly, PC2 explains the largest amount of the remaining variation after PC1 has been identified. The loadings for each PC and steroid are illustrated in Fig. 3. Most of the steroids contributed to PC1 and campesterol the most to PC2. The first component clearly separates the slurry type from the other two types, with almost all steroids contributing to the separation, even campesterol, though here, the difference is in the opposite direction (slurry less than the other two types). From the PC scores, the first three observations in each set of nine are clearly those with positive values of the first component; these are all slurry samples (right hand side of the biplot). There are two points in Fig. 3 in the upper right quadrant that are separate from the other slurry samples. These points have lower levels of 24-ethyl-coprostanol and much higher levels of campesterol than other slurry samples. Similarly, there are three soil samples in the lower left hand quadrant of Fig. 3 that are separate from the groupings. All these points have twice as much coprostanol than other soil samples. There is also a distinct sub-group of six points to the right of the left hand sub-set; these are the soil and both sample types from the fourth block (by the study field gate shown in Fig. 1). These samples all contain higher levels of steroids than the other blocks: 10 times as much 24-ethyl-coprostanol, 5 times as much epi-24-ethyl-coprostanol, and 3 times as much campestanol.

**Table 5:** PCA loadings (contributions) of each response variable to PC1 (91.67%) and PC2 (4.67%). The first 2 principal components explained 96.34% of the variance

Steroid	PC1	PC2
Campestanol	0.26858	0.05759
Stigmastanol	0.26842	0.05908
Epi-24-ethyl-coprostenol	0.26818	0.0696
24-Ethyl-coprostenol	0.2677	0.04183
β-Sitosterol	0.26745	0.04152
5ɑ-Cholestanol	0.26738	0.08819
Epi-Coprostanol	0.26701	0.06036
24-Ethyl-coprostanol	0.26548	0.04416
Stigmasterol	0.26405	− 0.01611
Coprostanol	0.26351	0.05387
5β-Campestanol	0.26346	− 0.02816
Epi-24-ethyl-coprostanol	0.26145	− 0.06337
Cholesterol	0.25299	0.03046
Epi-5β-campestanol	0.24878	0.14047
Campesterol	− 0.15687	0.97102

**Fig. 4:**
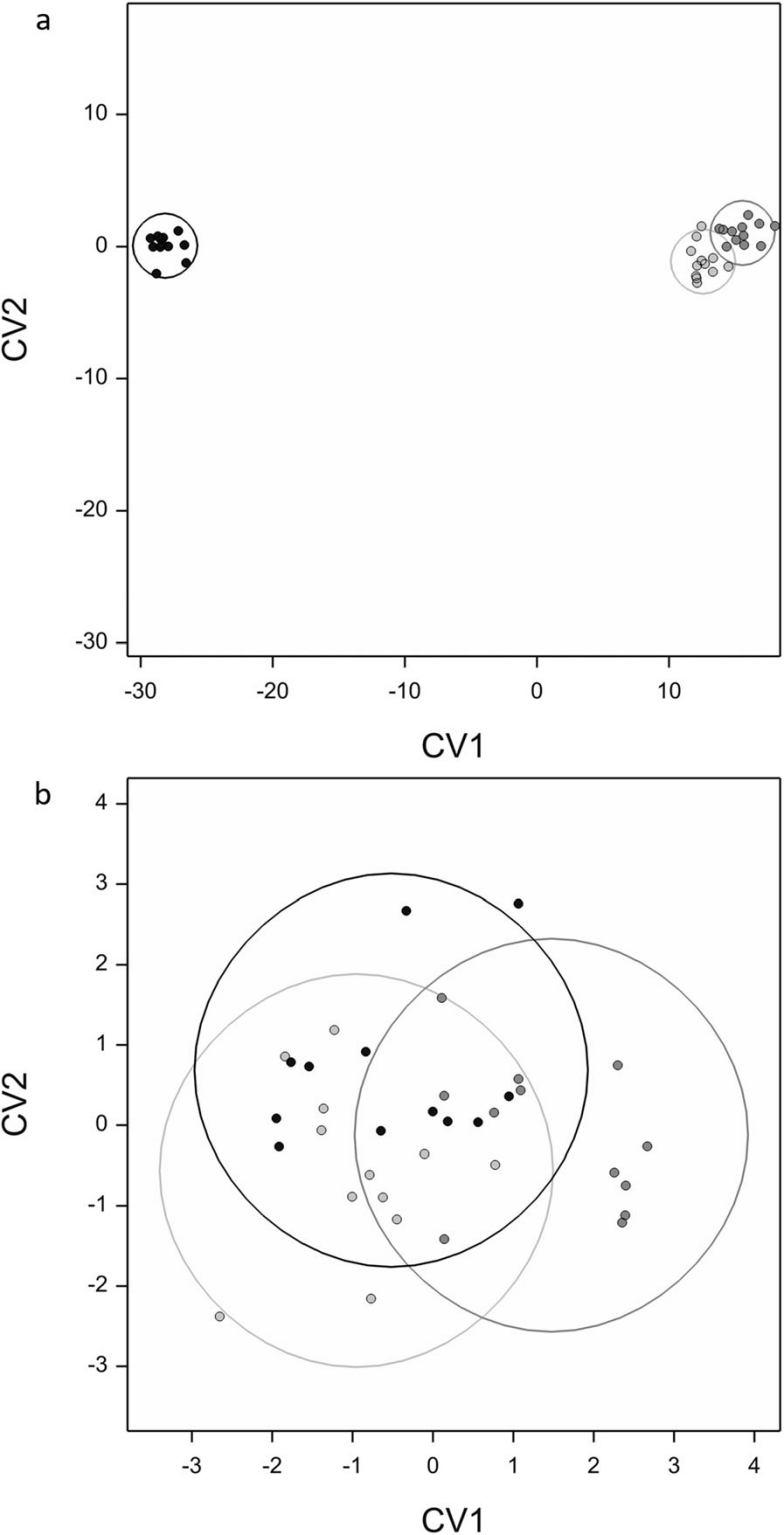
The canonical variate graphs for the steroidal analyses. The graph on the top (**a**) shows the significant groupings created when using type (both, light grey circle; slurry, black circle; soil, dark grey circle) as the grouping factor. The graph on the bottom (**b**) shows the slight, but not significant groupings when using the method as the grouping factor (ASE, black circle; Bligh and Dyer, dark grey circle; Soxhlet, light grey circle)

The results for the canonical variate analysis (CVA) regarding method suggest a weak discrimination between the three groups, and this is clearly seen in Fig. 4b; those for the types analysis (Fig. 4a) suggest a very strong discrimination, almost entirely associated with the difference between slurry and the other two types—in both cases, it is only the first component on which there is any discrimination. For the types analysis, there was a strong negative contribution of campestanol (with higher values apparently associated with higher values of soil and both) and a strong positive contribution of stigmastanol (where higher values are logically associated with the slurry type). Between PCA and CVA, it was possible to differentiate clearly between the slurry and soil/both sample types. However, whilst CVA shows a slight non-significant difference between soil and both, the combination of PCA and CVA were not capable of discriminating between the two sample types.

## Discussion

### Steroid Characterisation

Many of the steroids identified in this study have been previously identified in faecal samples (Leeming et al. 1996; Bull et al. 2002), although the total and individual steroidal content of agricultural animal faeces varies within the literature (Leeming et al. 1996; Tyagi et al. 2007; Prost et al. 2017). This could be due to differences in animal diet and there being no single methodology for their analysis which makes direct comparisons more problematic.

However, there is still a general trend that can be observed, such as predominating steroids and general steroidal content differences between species (Prost et al. 2017). These include coprostanol predominating human faeces compared with 24-ethyl-coprostanol dominating herbivorous faeces. Also, herbivorous faecal content is predominantly made up of phytosterols (stigmasterol, β-sitosterol, 24-ethyl-coprostanol, epi-24-ethyl-coprostanol, and 5α-stigmastanol). In the literature, these usually comprise between 64 and 89% of the total contents of steroids (Prost et al. 2017) which reflects the plant-based diet of herbivores. This corresponds with results in this study since 68% of the total steroids were phytosterols.

Some steroids are also widely found within the environment including some that are present in both animals and plants (cholesterol) and phytosterols found in higher plants (campesterol, stigmasterol and β-sitosterol) (Furtula et al. 2012; Murtaugh and Bunch 1967; Wen-Yen and Meinschein 1976). Accordingly, steroidal presence and values cannot be used alone. Leeming et al. (1996) determined the steroid distributions and concentrations in animal faeces and noticed variations in these steroid profiles depending on their source. As such, it was noted that ratios of these steroids can be used as more specific faecal indicators.

An additional complication is that these steroids are present in different ratios in many types of faeces. It has been established though, that by calculating the relative ratios of epi-coprostanol to 24-ethyl-coprostanol (ratio 1) (Leeming et al. 1996) or coprostanol to coprostanol + 24-ethyl-coprostanol (ratio 2) (Leeming et al. 1997; Harrault et al. 2019), it is possible to distinguish between human and herbivorous faecal matter (Leeming et al. 1996). The steroids 24-ethyl-coprostanol and epi-24-ethyl-coprostanol were indeed present in all samples, and as shown in Table 3 were on average highest in slurry samples and in similar amounts within soil and both types (though with both having marginally higher levels though not significantly). When using the ratios developed by Leeming et al. (1996, 1997), it was noted that for ratio 1, the average values were 0.1 for slurry, 0.1 for the soil, and 0.2 for both; for ratio 2, the corresponding ratios were 0.3 for slurry, 0.2 for soil, and 0.3 for both. With regard to ratio 1, values ~ 2.8 indicate human faeces, whereas 0–1.2 indicates animals (Leeming et al. 1997). This puts the slurry sample within the expected range (expected as this is cattle slurry) and the soil also (similarly unsurprising due to the past use of the study field). The both ratio was high, though not high enough to indicate human faecal matter. Exclusion of an unusually low 24-ethyl-coprostanol content reduced this ratio to 1.3 and closer to the expected value. For ratio 2, values < 0.38 indicate herbivore faeces, and values > 0.73 indicate human faeces. In this study, the average values for slurry, soil, and both all fall into the expected category indicating herbivorous faeces/contamination.

Regarding sample type, on average, slurry contained approximately 1000-fold the amount of steroids than the other two sample types. This high value corresponds with the existing literature, as well as the fact that the samples are predominated by 24-ethyl-coprostanol (Prost et al. 2017). This corresponds with the findings of past studies regarding the dominant steroids in ruminant faeces (Leeming et al. 1996; Bull et al. 2002; Gill et al. 2010).

### Extraction Method Comparison

As shown in Table 4, there was no significant difference (*P* < 0.05) in extraction method, except for one steroid (stigmastanol). This steroid also has the highest variance ratio (15.28) compared with the other steroids. Stigmastanol is a phytosterol, the product of the reduction of β-sitosterol and the biohydrogenation of stigmasterol by bacteria. However, as a 5ɑ-stanol, it is the product of biohydrogenation from microorganisms found within the environment, not within the gut of higher organisms. These are the 5β-stanols (Prost et al. 2017). As such, stigmastanol is not useful as a biomarker of cattle faeces, alone or in any ratio and so can be excluded from further method interpretation (at least for biomarker studies).

However, from the canonical variate analysis (Fig. 4b), there are visible groupings and separations between the methods, though this is very weak. It was observed that all of the methods had similarly high yields of steroids although ASE returned the highest yields in the majority of these cases, followed by Soxhlet and the BD. This indicates that ASE may be marginally more efficient, with BD being the least efficient. Previous work has, however, reported that ASE has marginally lower efficiencies than Soxhlet (Shen and Shao 2005) and that BD performs lower than Soxhlet also (Shah et al. 2006).

Whilst solid media were sampled in this study, it may be the need of other studies to sample other media. In previous studies (Isobe et al. 2004; Cordeiro et al. 2008; Gómez et al. 2012; Rontani et al. 2014), extraction has been completed using water filtered through glass fibre filters with the resultant suspended sediments being analysed. The collection and analysis of water samples for dissolved concentrations is not typically undertaken as previous studies have reported that > 95% of sterols are typically associated with the suspended sediment fraction of water samples (Isobe et al. 2002). In the aforementioned studies, the extraction method used was typically BD. However, in knowing that ASE is just as, if not more, efficient than BD for extracting lipids, there should be no technical difficulty in analysing suspended sediment media with ASE.

A recommendation from this study will be to determine the combination of resources required for lipid biomarker extraction and the nature of the solid media being investigated prior to full experimental work. Nevertheless, there is confidence from the robust analysis here that ASE can be used in the full characterisation of steroids for lipid biomarker research as appropriate and as an alternative in laboratories ordinarily using other methods. This is an important consideration, for example when laboratories are moving from one method to another and where some confidence in analysis continuity is required. Here, the results provide a full characterisation of steroid biomarkers for onward use in diffuse faecal pollution studies.

## Conclusions

Based on this study of three lipid extraction methods applied to slurry/soil samples, it can be concluded that there is no significant difference in the data generated by the extraction method used, regardless of the type of sample used, for each steroid extracted. This, being the case, choice of lipid extraction method for steroidal analyses becomes a choice based on economic factors, such as how resource (time/expense) intensive the method is and how much solvent is expended. It is clear, however, that based on these considerations, ASE is the more attractive method, as it is the quickest, least expensive, and consumes the smallest volume of solvents out of the three methods investigated.

## Data Availability

Data will be available on request.

## Abbreviations

BD: Bligh and Dyer
ASE: Accelerated solvent extraction
N: Nitrogen
P: Phosphorus
IMS: Industrial methylated spirit
DCM: Dichloromethane
TLE: Total lipid extract
BDS: Bligh and Dyer solvent
PCA: Principal component analysis
PC: Principal component
CVA: Canonical variate analysis
